# Re-Operation Rate for Breast Conserving Surgery Using Confocal Histolog Scanner for Intraoperative Margin Assessment—SHIELD Study

**DOI:** 10.3390/cancers17101640

**Published:** 2025-05-12

**Authors:** Michael P. Lux, Zlatna Schuller, Sara Heimann, Verena M. C. Reichert, Christian Kersting, Horst Buerger, Mariana-Felicia Sandor

**Affiliations:** 1 Department of Gynecology and Obstetrics, Women’s Hospital St. Louise, Paderborn, St. Vincenz-Krankenhaus, Husener Str. 81, 33098 Paderborn, Germanyfeliciasandor@outlook.de (M.-F.S.); 2 Institute of Pathology, Husener Strasse 46 c, 33098 Paderborn, Germany

**Keywords:** breast conserving surgery, confocal laser microscopy, intraoperative margin assessment, Histolog Scanner

## Abstract

**Introduction**: In breast conserving surgery (BCS), 15–40% of patients must undergo a second surgery (re-operation) due to post-surgical cancer-positive margins. Efficient intraoperative assessment of lumpectomy margins can reduce this rate. Classical methods like specimen radiography and ultrasound have limitations. The SHIELD study was conducted to prospectively quantify the reduction of the re-operation rate when the Histolog^®^ Scanner (HLS) confocal microscope is intraoperatively used by surgeons for the margin assessment. **Methods**: 50 patients undergoing BCS were enrolled and analyzed. Lumpectomy margins were intraoperatively assessed by surgeons with the HLS in addition to standard-of-care techniques. Detected positive margins triggered the excision of additional recuts during the same surgery. Subsequent re-operation and detection rates were compared to historical data and pathology gold standards, respectively. **Results**: The study population included 32% of patients with pure invasive cancer(s), 18% with pure DCIS and 50% with invasive cancer(s) mixed with DCIS. The overall mean age was 63.56. All population features were statistically similar to the historical control (*p* > 0.1). Notably, 80.95% sensitivity and 99.53% specificity for breast cancer detection at the margin were intraoperatively achieved by the surgeons using the HLS. The re-operation rate in SHIELD was 10% (5/50) while the historical control was 30% (12/40) corresponding to a 67% reduction (*p* = 0.016). Notably, 17/21 positive margins were intraoperatively identified with the HLS while 4/21 were detected with standard-of-care techniques. **Conclusions**: The intraoperative use of the Histolog Scanner confocal microscope provides a significant increase in detection rates of lumpectomy positive margins resulting in a substantial reduction in the re-operation rate, while preserving specimen integrity without impact on histopathology assessment.

## Simple Summary

Intraoperative margin assessment in breast conserving surgery with classical methods (radiography and ultrasound) has limitations resulting in 15–40% of patients that must undergo a second surgery (re-operation). The SHIELD study was conducted on 50 patients to quantify the reduction of the re-operation rate when the Histolog^®^Scanner confocal microscope is used to assess the presence of positive margins and excise additional recuts during the same surgery. 80.95% sensitivity and 99.53% specificity for breast cancer detection at the margin were achieved by the surgeons using the Histolog^®^Scanner. The re-operation rate in SHIELD was 10% (5/50) representing a 67% reduction (*p* = 0.016) when compared to 30% of the historical data. 17/21 and 4/21 positive margins were identified by surgeons using the Histolog^®^Scanner and the standard-of-care techniques, respectively. The intraoperative use of the Histolog^®^Scanner confocal microscope increased cancer detection rates resulting in a significant and clinically relevant reduction in the re-operation rate.

## 1. Introduction

Breast cancer is the most frequently diagnosed type of cancer worldwide in women and is the leading cause of cancer-related deaths among women globally leading to 2.3 million new cases of breast cancer and 665,000 related deaths in 2022 [1]. A substantial proportion of breast cancer patients are diagnosed at an early stage allowing consideration of a breast conserving surgery (BCS) approach [2]. In addition, DCIS has significant clinical relevance due to its high incidence and surgical challenges. The surgical technique aims to eliminate breast cancer lesions entirely while preserving as much healthy tissue as possible. BCS should yield clear postoperative margins following the surgical resection, allowing for the continuation of subsequent care measures in breast conserving treatment, such as radiotherapy. Notably, 15–40% of patients who undergo BCS face the need for a subsequent operation, called re-operation, due to incomplete lesion removal and positive or close margins, especially in the case of DCIS or accompanying DCIS [3,4,5]. Positive margins correlate with significantly increased rates of local recurrence [6,7]. Re-operation due to positive margins leads to various challenges, including delayed adjuvant therapy, negative cosmetic outcomes, and significant psychological and financial burdens on the patients as well as resource and financial impacts on health care systems.

During BCS, visual assessment and palpation inspection are usually performed by breast surgeons. In addition, several intraoperative techniques are available, although their evidence and practical applications can vary significantly. Established methods include pathological techniques like frozen section, along with imaging techniques such as specimen radiography and/or intraoperative ultrasound. Frozen section analysis typically shows strong diagnostic accuracy; however, it is not commonly implemented because of the resource-intensive nature of this pathological technique, along with slow turnaround times, disruptions to surgical workflow, and associated costs [8,9]. Specimen radiography is a well-established radiological technique that is routinely used in many hospitals to confirm tumor localization in the excised specimen. It can be used for margin assessment, but it shows limitations for small tumor infiltrates, lobular cancer and DCIS that is indirectly correlated with the presence of microcalcifications [10]. The practice of intraoperative ultrasound is increasing in BCS and is providing higher diagnostic accuracy than specimen radiography. However, it can only be applied to detectable lesions [11,12]. To address the limitations of these conventional approaches, innovative tools such as optical spectroscopy, micro-CT, and radiofrequencies are currently under evaluation, but none have been adopted by the community [13].

Confocal laser microscopy has been known for decades in the biomedical field as an efficient method for fresh tissue imaging. Confocal laser scanning microscopes (CLSM) were specifically designed for clinical applications [14,15,16]. The Histolog^®^ Scanner (HLS), a medical device based on the CLSM approach, has been developed for intraoperative and pathology applications with a wide field-of-view (4.8 × 3.6 cm), high resolution (2 µm) and speed (<1 min per image). The HLS is able to provide en-face visualization of the scanned surface (whole margin assessment) with histological details similar to frozen sections [8]. The use of the HLS for efficient cancer detection in specimen margins has been reported in skin, prostate and lymph node tissues for a few years [17,18,19,20]. In breast tissue, the initial feasibility study has shown that the use of the HLS allows accurate detection of breast cancer in breast biopsies by pathologists [21]. Another study on regions of interest in HLS images has shown that trained breast surgeons are also able to recognize breast cancer with high accuracy [22]. Assessment by breast surgeons is of particular interest to overcome the shortage of pathology resources that can be observed in some clinical institutions. Several observational studies have been performed with the HLS on lumpectomies in an operating room setting including the POLARHIS study performed in our center. They showed that both invasive ductal cancer (IDC), non-specific type (NST), invasive lobular cancer (ILC) and DCIS cancer lesions can be efficiently identified in lumpectomy margins by pathologists but also by trained breast surgeons [23,24,25]. Detection rates in these studies were promising and it is suggested that the sensitivity could be improved with advanced training material for physicians. Recently, an extensive atlas of breast lumpectomy images with the HLS has been released [26] as well as a training program for physicians to identify breast cancer in HLS images of lumpectomy margins [27]. Following our previous experience with the HLS in POLARHIS and the availability of these supporting materials, the SHIELD study includes for the 1st time a prospective use of the HLS by surgeons in real time during surgery to perform margin assessment in order to monitor its impact on the re-operation rate and evaluate clinical detection performance.

## 2. Methods

### 2.1. Patient Population

From October 2023 to March 2024, 54 patients with breast cancer and/or DCIS treated with primary breast conserving surgery and without neoadjuvant treatment were screened and gave consent to participate in the study in accordance with study protocol approved by the Ethics committee of the Ärztekammer Westfalen-Lippe and the WWU Münster on 26 June 2023 (No. 2023-385-f-S) and registered on the NIH database (NCT05946759). Notably, 2 patients did not fulfill the study selection criteria and 2 were presenting incomplete collection of data resulting in a population of 50 patients included in the analysis.

The historical control group comprises 40 subjects from the POLARHIS study (Ethics Committee Münster approval 2020-578-f-S). POLARHIS was an observational study performed in the same clinical center and surgical team, following the same patient selection criteria which included an HLS imaging performed after patient closing without impact on clinical outcome [23].

### 2.2. Equipment

Surgical specimens were imaged using the Histolog Scanner (SamanTree Medical, Lausanne, Switzerland) a CE-marked, and FDA 510k cleared, wide field-of-view confocal laser scanning microscope specifically designed for high-resolution imaging of large biological specimens in the medical setting such as the operating theater. To enhance specimen contrast, the Histolog Dip, an acridine orange-based fluorescent dye solution (SamanTree Medical, Lausanne, Switzerland) is used on the tissue specimen. The scanner produces high-quality fluorescence images without the need for additional post-processing. It is ready for use within seconds of being powered on, with no calibration or parameter adjustments needed by the user. High-resolution images of the specimen surface are captured in less than 1 min per image, enabling immediate detailed visualization of tissue morphology down to the level of cell nuclei.

### 2.3. Surgeon Training

Surgeons completed the device training provided by the manufacturer to operate the HLS. Then each surgeon imaged 10 lumpectomy specimens without any impact on the patient treatment prior to study initiation to feel comfortable in the generation of good Histolog images in the demanding setting of the operating theater. Surgeons have followed a training program for 6 h to be able to screen and understand Histolog image content of breast tissue as described by Guani and Cattacin [27].

### 2.4. Margin Assessment

During the surgery, the surgeon excised the lumpectomy specimen following standard-of-care surgical practices of the certified breast cancer center and using cold blades and scissors. The specimen was oriented using surgical threads and carefully blotted with surgical pads to remove excess blood. Next, the specimen was stained for 10 s with the Histolog Dip fluorescent solution, rinsed briefly with saline solution, and gently blotted again with a clean surgical pad. The specimen was then positioned over the imaging window, and images of the specimen were collected in en-face orientation. All lateral specimen margins were always imaged with the HLS sometimes complemented with frontal or dorsal margins if relevant for surgical guidance. Histolog images were assessed in real time by the surgeon and the time to perform the overall Histolog assessment was monitored. In addition, conventional techniques (visual inspection, palpation, ultrasonography and/or radiography of the specimen) were performed at the discretion of the surgeon. Positive margin(s) identified in the Histolog images and/or with conventional techniques triggered the excision of additional tissue recuts during the same surgery. After margin assessment, surgical specimens underwent histopathology assessment following standard-of-care.

### 2.5. Statistical Analysis

A descriptive statistical analysis of all 50 patients was performed. All continuous variables were displayed as mean and standard deviation, whereas categorical values were displayed by frequencies and percentages, when appropriate. All statistical analyses were two-sided tests with a 5% significance. All statistical analyses were performed using SAS version 9.4 (SAS Institute Inc., Cary, NC, USA). Statistical comparisons between patient populations in the study-treated group and historical control group were evaluated with Fisher’s Exact Test.

## 3. Results

### 3.1. Study Collective

50 patients were enrolled and analyzed with a mean age of 63.56 years (47–84 years). No adverse events were reported intraoperatively or through the final pathology assessments that included H&E staining and immuno-histochemistry techniques. Patients’ lesions were palpable for 25/50 patients with an average size of 1.81 ± 0.92 cm while the other half of the patients (25/50) patients were presenting non-palpable lesions with an average size of 1.02 ± 0.60 cm. Notably, 32% of the patients were presenting with pure invasive tumors (22% of non-lobular carcinoma and 10% of ILC) while DCIS was found in 68% of the patients either pure (18%) or associated with invasive tumors (50%). The mean length, width and thickness of the lumpectomy specimens were 5.80 cm, 4.27 cm and 3.67 cm, respectively. SHIELD and the historical control groups were not statistically different for patient and tumor characteristics (Table 1).

### 3.2. Intraoperative Assessments

Standard-of-care conventional techniques of the certified breast center (palpation and visual inspection, ultrasound and/or radiography) were performed at the discretion of the surgeon depending on the case. Usage of the conventional techniques in SHIELD patients and historical control groups was very similar. Palpation and visual inspection were used in 96.0% and 92.5% of the SHIELD group and the control group, respectively. Ultrasound was used in approximately 2/3 of the patients (68.0% and 67.5% for SHIELD and the retrospective control group) while radiography was applied to approximately 1/3 of the patients (40.0% and 32.5% for SHIELD and the control group), see Table 2.

**Figure 1:**
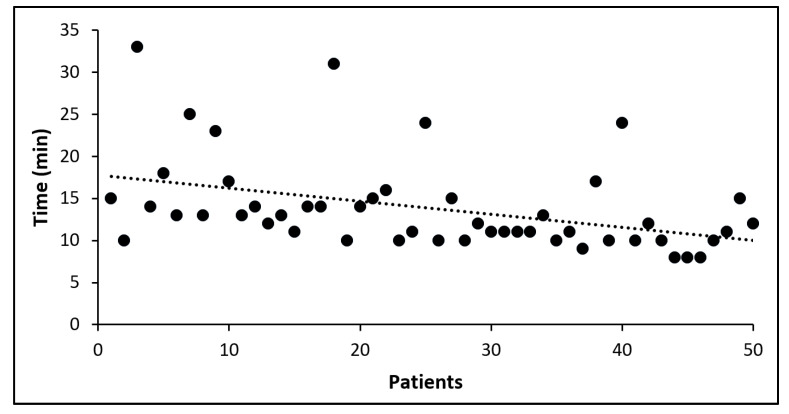
Mean time per patient for the margin assessment with the HLS (dotted line: linear regression).

In addition to conventional techniques, the HLS was used by surgeons to assess specimen margins for all patients. All the generated images were judged as assessable by the surgeons. The average time to perform an assessment of all lateral margins with the HLS was 13:50 ± 5:29 min (6:31 ± 1:40 min for sample processing and image acquisition, and 7:19 ± 4:53 min for image analysis), which corresponds to 2:57 ± 1:21 min per margin (Table 3). Times related to HLS assessment decreased during the study with overall mean times per patient around 17 min for the first patients and the mean value of 11 min for the last patients (Figure 1). Since the overall time for imaging remained constant throughout the study, this decrease is probably due to a reduction of the time dedicated to image analysis by surgeons as a result of learning and experience.

In order to quantify the intraoperative breast cancer detection performance of the surgeons, margin status determined during surgery using the HLS and the conventional standard-of-care techniques were collected and compared to the final diagnosis established by the pathologist on paraffin-H&E microscopic slides after the surgery without knowing the output of the intraoperative assessment. These comparisons determine if surgeon assessments were true positive, true negative, false positive or false negative for the margin status. The corresponding sensitivity, specificity, accuracy, positive predictive value and negative predictive values of breast cancer detection with HLS were 80.95%, 99.51%, 97.80%, 94.44% and 98.09%, respectively. Breast cancer detection with conventional standard-of-care techniques demonstrated good specificity but approximately 4 times lower sensitivity than the HLS. Sensitivity, specificity, accuracy, positive predictive value and negative predictive values provided by the conventional standard-of-care techniques were 19.05%, 97.27%, 93.60%, 25.00% and 96.18%, respectively (Table 4).

**Figure 2:**
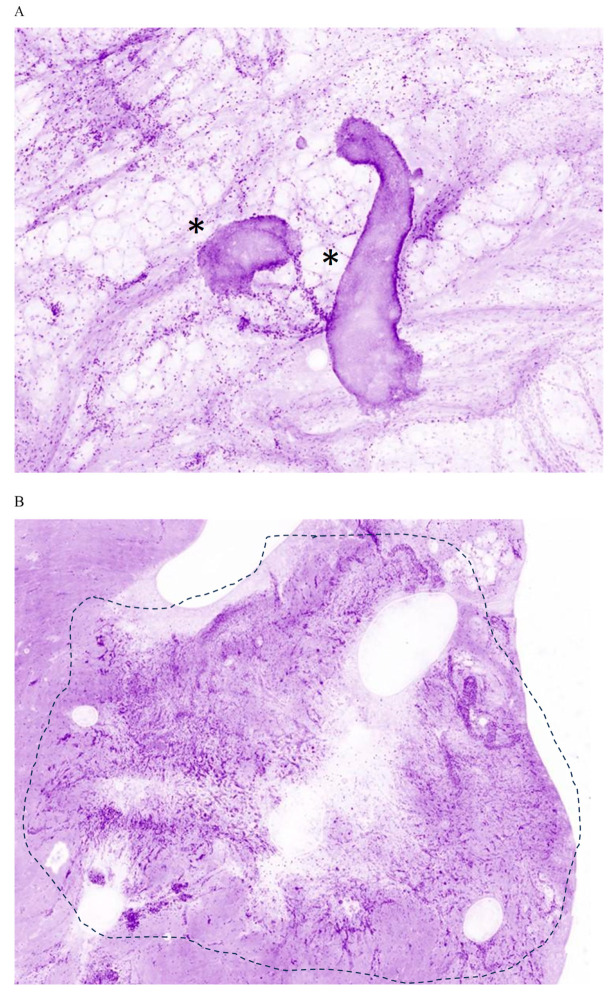

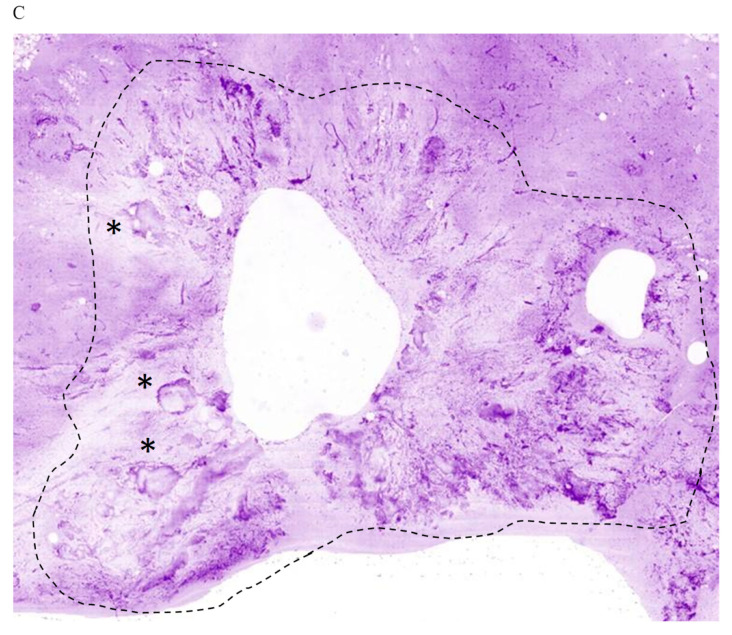
HLS images of breast cancer-positive margins (crops). (**A**) DCIS lesions (*) seen as solid assemblies of cancer cells presenting an epithelial pattern and surrounded by fatty tissue (high magnification). (**B**) ILC-positive margin. Cancer is seen within the dotted line annotation with tumor nests and Indian files of cancer cells infiltrating into connective tissue (mid magnification). (**C**) IDC/NST positive margin. Cancer is seen within the dotted line annotation as a stellar pattern of tumor nests and Indian files of cancer cells complemented with solid DCIS components (*) (mid magnification).

The use of the HLS allowed the surgeons to detect IDC NST, DCIS and ILC lesions in lumpectomy margins (Figure 2). Overall, the use of the HLS allowed to detect 80.9% (17/21) of positive margins while the conventional techniques were able to detect 19% (4/21) of positive margins. Notably, 90.9% of DCIS positive margins were detected using the HLS while the conventional techniques were able to detect 10 times less (9.1%). The use of the HLS allowed the detection of 62.5% of IDC/NST positive margins and the conventional techniques allowed the detection of 37.5%. Finally, all ILC-positive margins were detected with the HLS while none of them were detected with the conventional techniques (Table 5). All the positive margins identified using conventional techniques were also identified using the HLS.

### 3.3. Re-Operation Rates

There were 10% (5/50) re-operations in the SHIELD patient population with the HLS assessment in addition to standard-of-care techniques. All the re-operations occurred in patients with a breast cancer type composed of DCIS mixed with non-lobular invasive cancers (here exclusively IDC/NST). In the historical control group using only standard-of-care intraoperative margin assessment techniques, 30% (12/40) of patients had to undergo a re-operation [23]. The use of the HLS for intraoperative margin assessment resulted in a statistically significant reduction of the re-operation rate from 30% to 10% (*p* = 0.016) representing a relative reduction of 66.6% (Table 6).

Overall, there were 12/50 patients that had positive margin(s) on the primary surgical specimen according to the final pathology assessments. 4/12 patients had at least one positive margin missed during the intraoperative assessments and these patients had to undergo a re-operation to remove the remaining cancer lesions. Notably, 7/12 patients obtained all their positive margins accurately detected by the intraoperative margin assessments (5/12 patients exclusively detected exclusively with the HLS and 2/12 patients, detected with both the HLS and standard-of-care techniques). The subsequent additional recuts intraoperatively performed were sufficient to remove all the remaining cancer and these patients did not have to undergo a re-operation. In one additional patient with all her positive margins accurately detected with the HLS, the subsequent recuts performed intraoperatively were not sufficient to remove all the remaining cancer and this patient still had to undergo a re-operation (Table 6).

## 4. Discussion

Providing accurate intraoperative assessment of lumpectomy margins is expected to achieve negative surgical margins at primary surgery and reduce the need to perform a second conserving surgery for a complete removal of breast cancer from the patient (re-operation). Several methods are used to reduce the presence of cancer-positive margins and the associated re-operations in BCS. These methods include gross examination of the lumpectomy specimen, frozen sections, touch prep analysis, intraoperative specimen radiography, and intraoperative ultrasound, as well as investigational tools [8,9,10,11,12,13].

Here we present the 1st evaluation of the Histolog Scanner (HLS) usage to intraoperatively assess lumpectomy margins and treat the patient in real time. The device was easy to integrate in the operating room with a quick and simple setup. In the present study, the intraoperative assessment of all lumpectomy margins was performed by breast surgeons with the HLS in approximately 13 min making it compatible with the surgical timings. The device is user-friendly, and we expect that its operation and image acquisition could be delegated to trained surgical assistants or OR staff under surgeon supervision. The surgeon can then continue with other parts of the operation in parallel, such as the sentinel node excision. The use of the device has not created any adverse events during and after the surgery and has not impacted the final pathology assessment as previously reported [17,18,19,20,21,22,23,24,25,26,28]. All the produced images were of sufficient quality to allow analysis of the margin content. Our standard-of-care surgical practices include a very limited use of electrocautery for specimen excision. In our opinion, this practice allowed us to achieve excellent quality of the images as already observed in our previous study with the HLS [23]. In studies with recurrent usage of electrocautery, its usage was reported to stiffen the specimen and create cautery artifacts resulting in limited image readability [24,25]. The use of electrocautery, even independently of the HLS, impairs pathological assessment and it is generally recommended to use it with caution for specimen excision.

Breast cancer detection in HLS images of lumpectomy margins was achieved by surgeons with sensitivity and specificity values of 80.9% and 99.5%, respectively, providing values close to FSA [8]. In previous studies, sensitivity values achieved by surgeons ranged between 27.27% to 37.5% [23,24,25]. The substantial improvement measured in the present study is attributed to the training program followed by the surgeons [27] in contrast to the limited introductory materials that were used in the previous research. Such sensitivity (80.9%) complemented with very good positive predictive values (>94%) is considered sufficient to use this approach in a routine clinical setting especially when compared to the standard-of-care techniques that provide a limited level of detection with 4 times lower sensitivity values (19.05%) in the same population. The rate of false positive assessments in the SHIELD study was only 1/235 assessments (0.42%) with the HLS providing very high specificity (99.5%) and negative predictive values (98.1%). It shows that the approach with the HLS is highly compatible with surgical procedures intended to be conservative such as the BCS. As a comparison, standard-of-care techniques were providing 6× more false positives but still at an acceptable level (12/461 = 2.60%). In addition, the study has also shown that the use of the HLS allowed the detection of all the positive margins identified with standard-of-care techniques. There were no positive margins that were detected with standard-of-care techniques and missed by surgeons using the HLS. This is a useful finding for centers willing to reorganize their intraoperative assessment practices. 

As defined in the protocol, the intraoperative assessment of the recut(s) was not included in the study. One patient had a successful intraoperative assessment with the HLS but had to undergo a re-operation for BCS due to insufficient tumor removal in the recuts. We could hypothesize that the assessment of these recuts using the HLS would have been able to further guide the surgeon and achieve a complete removal of the tumor at initial surgery increasing the benefit of using the HLS in the BCS context and resulting in even further reductions in re-operation rates. There is also the possibility of further developing artificial intelligence within the system. In the future, for example, AI could present the surgeon areas of particular interest and thus further optimize the assessment as previously suggested [22].

The occurrence of the positive margin and its associated re-operation rates can be reduced in some cases by performing a larger excision with increased distance between the tumor core and specimen margins. The main specimen sizes of the SHIELD study population are not statistically different from the specimen sizes of the control group (*p* > 0.1). Therefore, the reduction in the re-operation rate observed for SHIELD populations is expected to be linked to increased breast cancer detection rates while maintaining similar surgical specimen dimensions from a conservative surgical approach.

Interestingly, the use of the HLS allowed the detection of all main types of breast cancer in lumpectomy margins. Surgeons were able to detect the positive margins in the majority of patients with DCIS (10/11) and ILC (2/2) types. This is a great advantage since these lesions are usually non-palpable and they constitute a challenge in which surgeons are sorely lacking support for these patients [29,30,31]. The detection rate for palpable invasive carcinoma is substantial (5/8) but lower than non-palpable lesions. This could be hypothesized with a retraction of the dense tumor core upon surgical excision creating an invagination of the specimen that will not be visualized in HLS images as previously suggested [25].

Limitations of this study include a limited sample size and a non-randomized design. The control group used in this study is not composed of data prospectively collected for the study but is coming from historical data collected along the Polarhis study. Finally, patients with neoadjuvant treatments were also excluded from the study and demonstration of this specific population may be of interest to the community.

## 5. Conclusions

The prospective SHIELD study is the first interventional study using the Histolog Scanner in BCS reported in the literature. It demonstrates that the use of the HLS by surgeons for intraoperative margin assessment of breast lumpectomy specimens significantly reduces a clinically relevant number of re-operations due to cancer-positive margins from 30% to 10%, a 67% reduction, while maintaining the conservative aspect of the surgical approach. Use of the HLS demonstrated good Sensitivity for all breast cancer types in a timeframe compatible with operating room constraints. It preserves specimen integrity without impact on further histopathological assessments. SHIELD confirms the promising results from the observational studies previously published.

## Figures and Tables

**Table 1:** Patient and Tumor Characteristics.

Parameter	SHIELD	Control	*p*-Value
n Patients	50	40	-
Mean Age (SD)	63.56 (10.32)	62.60 (9.56)	0.6517
Breast cancer type			0.9283
- Invasive carcinoma	32.0% (16/50)	32.5% (13/40)	
- DCIS	18.0% (9/50)	15.0% (6/40)	
- Invasive with accompanying DCIS	50.0% (25/50)	52.5% (21/40)	
Invasive Subtypes			0.9545
- Lobular carcinoma	10.0% (5/50)	10.0% (4/40)	
- Non-lobular carcinoma	72.0% (36/50)	75.0% (30/40)	
Palpable Lesions	50.0% (25/50)	45.0% (18/40)	0.6370
Size of Palpable Lesions [cm]	1.81 (0.92)	1.62 (0.63)	0.4636
Size of Non-Palpable Lesions [cm]	1.02 (0.60)	1.10 (0.85)	0.7138
Mean Specimen Sizes (SD) [cm]			
- Length	5.80 (2.83)	5.97 (1.48)	0.7201
- Width	4.27 (2.26)	4.56 (1.36)	0.4541
- Thickness	3.67 (3.06)	3.10 (2.18)	0.3113

**Table 2:** Intraoperative Assessments with standard-of-care conventional techniques performed to assess lumpectomy margins.

	Palpation and Visual Inspection	Ultrasound	Radiography
SHIELD	48/50 (96.0%)	34/50 (68.0%)	20/50 (40.0%)
Control	37/40 (92.5%)	27/40 (67.5%)	13/40 (32.5%)

**Table 3:** Mean time of intraoperative assessments of lumpectomy margins with the HLS. Standard deviation is shown between brackets.

	Mean Time per Patient (SD)	Mean Time per Margin (SD)
Overall	13 min 50 s (5 min 29 s)	2 min 57 s (1 min 21 s)
Image acquisition	6 min 31 s (1 min 40 s)	1 min 23 s (0 min 29 s)
Image analysis	7 min 19 s (4 min 53 s)	1 min 34 s (1 min 03 s)

**Table 4:** Intraoperative cancer detection performance of surgeons in lumpectomy margins using the HLS and the conventional standard-of-care techniques. Notably, 95% Confidence Interval is shown between brackets.

Parameter	HLS N = 227	Standard-of-Care Techniques N = 461
Sensitivity	80.95% (64.16–97.75%)	19.05% (2.25–35.84%)
Specificity	99.51% (98.57–100.0%)	97.27% (95.75–98.79%)
Accuracy	97.80% (95.89–99.71%)	93.71% (91.49–95.93%)
PPV	94.44% (83.86–100.0%)	25.00% (3.78–46.22%)
NPV	98.09% (96.23–99.94%)	96.18% (94.40–97.96%)

**Table 5:** Type of breast cancer found on positive lumpectomy margins by the final assessment and intraoperative detection with HLS and standard-of-care conventional techniques.

**Cancer Type**	**Final Assessment**	**HLS**	**Standard-of-Care**
DCIS	11	10/11 (90.9%)	1/11 (9.1%)
IDC/NST	8	5/8 (62.5%)	3/8 (37.5%)
ILC	2	2/2 (100%)	0/2 (0%)
TOTAL	21	17/21 (80.9%)	4/21 (19.0%)

**Table 6:** Re-operation Characteristics of SHIELD and control group populations.

	Re-Operation Rates		
	SHIELD	Control	*p*-Values
Re-operation Rate (95% CI)	10.0% (5/50) (3.3–21.81%)	30.0% (12/40) (16.56–46.53%)	0.016
**Cancer type of patients with Re-operations**			
DCIS	0.00% (0/50)	10.0% (4/40)	
Non-lobular invasive cancer	0.00% (0/50)	0.00% (0/40)	
Non-lobular invasive cancer with DCIS	10.0% (5/50)	15.0% (6/40)	
Lobular invasive cancer	0.00% (0/50)	5.0% (2/40)	
**Positive margins in Patients and Re-operations**			
Patients with positive margins	12/50	13/40	
Patients without a Re-operation due to efficient intraoperative assessment	7/12	1/13	
- HLS only	5/12	Not Applicable	
- Standard of care only	0/12	1/13	
- HLS and Standard of Care	2/12	Not Applicable	
Patients with Re-operation due to positive margin(s) missed by intraoperative assessments	4/12	12/13	
Patients with Re-operation due to insufficient recut(s) accurately triggered using HLS	1/12	Not Applicable	

## Data Availability

The datasets generated and analyzed during the current study are available from the corresponding author upon reasonable request.
